# Evaluation of sestrin 2 and tribbles homolog 3 levels in obese and nonobese women with polycystic ovary syndrome

**DOI:** 10.55730/1300-0144.5738

**Published:** 2023-12-04

**Authors:** Ayşegül ÇATAL, Evrim Ebru KOVALAK

**Affiliations:** 1 Department of Medical Biochemistry, University of Health Sciences, Bağcılar Training and Research Hospital, İstanbul, Turkiye; 2 Department of Obstetrics and Gynecology, University of Health Sciences, Bağcılar Training and Research Hospital, İstanbul, Turkiye

**Keywords:** Insulin resistance, obesity, polycystic ovary syndrome, sestrin 2, tribbles homolog 3

## Abstract

**Background/aim:**

This study was designed to evaluate the relationship of two new biomarkers [tribbles homolog 3 (TRB3) and sestrin 2 levels], which were previously associated with obesity, with metabolic parameters in obese and nonobese women with polycystic ovary syndrome (PCOS).

**Materials and methods:**

This cross-sectional case control study was conducted between September 2017 and August 2019 in the gynecology department of a tertiary referral hospital. The values of the plasma sestrin 2, TRB3, insulin, fasting plasma glucose, lipid profile, and homeostasis model assessment of insulin resistance (HOMA-IR) were compared in 90 obese women with PCOS (BMI > 30), 90 women with nonobese PCOS (BMI < 30), and 90 control patients (BMI < 30).

**Results:**

The mean age of the study group consisting of all PCOS patients (26.11 ± 4.64 years) and the mean age of the control group (26.3 ± 4.4 years) were statistically similar (p = 0.239). The serum sestrin 2 values of the obese PCOS group were found to be statistically significantly lower than the control and non-obese PCOS groups (p = 0.001, p = 0.0001), while the sestrin 2 values of the nonobese PCOS group were found to be statistically significantly lower than the control group (p = 0.0001). The TRB3 values of the control group were found to be statistically significantly lower than the obese and nonobese PCOS groups (p = 0.0001), while the TRB3 values of the nonobese PCOS group were found to be statistically significantly lower than the obese PCOS group (p = 0.0001). A negative correlation was observed between the sestrin 2 level and BMI (r = −0.272 p = 0.0001), insulin (r = −0.261 p = 0.0001), and HOMA-IR levels (r = −0.250 p = 0.0001). A positive correlation was observed between the TRB3 values and TG (r = 0.248 p = 0.0001), and LDL-C values (r = 0.235 p = 0.0001).

**Conclusion:**

According to the findings in this study, low sestrin 2 and high TRB3 levels may be related to impaired metabolic status in the obese PCOS group. Thus, it may be promising for the development of treatment of PCOS and associated metabolic disorder in the future.

## 1. Introduction

Polycystic ovary syndrome (PCOS) is one of the most common (8%**–**13%) but least understood endocrinopathies in women of reproductive age, characterized by hyperandrogenemia, hyperinsulinemia, hypothalamic-pituitary-ovarian axis dysfunction, and irregular secretion of adipokines from adipose tissue. Moreover, PCOS can cause female subfertility [1,2]. Insulin resistance (IR), obesity, metabolic syndrome, cardiovascular disease, type 2 diabetes and hyperlipidemia are common in women with PCOS [3,4]. A clear gene disorder causing PCOS has not been identified despite there being a number of environmental factors, such as obesity, that contribute to PCOS. Hyperinsulinemia plays a fundamental role in the pathogenesis of PCOS [4]. Approximately 50%–80% of women with PCOS have insulin resistance, and 30%–70% are obese or overweight. The presence of obesity and IR significantly exacerbates both the clinical and laboratory expression of PCOS [5**–**7]. Serum AMH is synthesized by early antral follicles in women and its levels are closely related to the number of early antral follicles. The overproduction of AMH through the accumulation of preantral follicles is attributable to impaired folliculogenesis, hyperandrogenemia and insulin resistance in PCOS, and there is evidence that excess AMH may be associated with the severity of the syndrome [3].

It has been suggested that disorders related to autophagy, a stress-induced lysosome-mediated degradation process, cause insulin resistance. In addition, dysregulation of autophagy was observed in the ovarian granulosa cells and endometria of women with PCOS [8]. It is also thought to be associated with increased oxidative stress and insulin resistance in PCOS [9]. Oxidative stress occurs when reactive oxygen radicals and reactive nitrogen species increase [10]. Oxidative stress is thought to cause hyperandrogenemia in PCOS by inhibiting various proteins [11]. In a study of women with PCOS, circulating oxidative stress markers were found to be high [12].

Sestrin 2 is considered an antioxidant protein that protects against oxidative stress, reactive oxygen species and cardiovascular diseases by regulating autophagy [13,14]. Sestrin 2 reduces IR through the regulation of glucose and lipid homeostasis [15]. One study in patients with PCOS reported that plasma sestrin 2 levels were significantly lower than those in healthy controls [8]. However, in another recent study by Beskel et al., sestrin levels were found to be high as an indicator of oxidative stress in women with PCOS [16]. Tribbles homolog 3 (TRB3) is a mammalian homologue of the Drosophila Tribbles gene secreted from tissues such as the liver, adipose tissue, skeletal and cardiac muscle under metabolic stress induction, also called NIPK (neuron cell death-inducible protein kinase) [17,18]. A study in Chinese women found that TRB3 Q84R polymorphism was associated with obesity and especially glucose metabolism, but not with PCOS and IR [19]. This study was designed to examine the relationship of these two new biomarkers (sestrin 2 and TRB3) associated with metabolic disorders and obesity in PCOS to shed light on further studies on etiopathogenesis and treatment modalities.

## 2. Materials and methods

This cross-sectional case control study was conducted in a tertiary referral hospital gynecology outpatient clinic between September 2017 and August 2019. The study was approved by the Local Ethics Committee of our hospital (Clinical Research IRB No: 2017/596). Every participant was informed, and their written consent was obtained.

### 2.1. Patient characteristics

Women between the ages of 18 and 45 who were diagnosed with PCOS using the Rotterdam criteria were included in the study [20]. The following criteria were considered: polycystic ovarian morphology (diameter 2–9 mm, ≥ 12 follicles in both ovaries) by transvaginal ultrasound, oligo/amenorrhoeic cycles (menstrual cycle >35 days/amenorrhoea > 3 months), clinical (Ferriman-Gallwey score > 12) [21] or biochemical (total testosterone level > 2.6 nmol/L) hyperandrogenism. PCOS is diagnosed when two of these three main features are identified.

### 2.2. Physical measurements and laboratory evaluation

Height and weight measurements were made with the same digital electronic device. Body mass index (BMI) was calculated by dividing the body weight (kg) by the height (m) squared. Two-hundred-and-eighteen women were diagnosed with PCOS. Thirty-eight women were excluded from the study due to additional systemic diseases and drug use. Women with BMI ≥ 30 kg/m^2^ (n = 90) were considered to be the obese PCOS group, and women with BMI < 30 kg/m^2^ (n = 90) were considered to be the nonobese PCOS group. The control group (n = 90) consisted of women with BMI < 30 kg/m^2^, in the same age group, with normal ovarian function, who applied at the outpatient clinic for routine gynecological examinations, and did not have serious gynecological disorders. In the early follicular phase of every woman’s menstrual cycle, blood samples were taken from the antecubital vein between 8:00 am and 10:00 am after 12 h of fasting. The blood samples were centrifuged at 4000 g for 15 min and the serum samples were separated and stored in a deep freezer at −80 °C in Eppendorf tubes until the day when all samples were to be studied at the same time. The serum glucose, insulin, total cholesterol (TC), triglycerides (TG), HDL-cholesterol (HDL-C), LDL-cholesterol (LDL-C), Anti-Mullerian hormone (AMH), sestrin 2 and TRB3 levels of every patient were measured. IR was calculated using the serum insulin formula (μIU/mL × fasting plasma glucose (mg/dL)/405) (homeostatic model assessment of IR, HOMA-IR) [22]. The exclusion criteria were breastfeeding or pregnancy, endocrine diseases, oncological diseases, and the use of hormonal drugs including contraceptives, within three months.

The serum sestrin 2 concentrations were measured using a sandwich enzyme linked immunosorbent assay (ELISA) kit (Human SESN2 ELISA Kit, SunRed, Shanghai, China; Catolog no. 106-201-12-4926). The serum TRB3 concentrations were measured using a sandwich enzyme linked immunosorbent assay (ELISA) kit (Human TRB3 ELISA Kit, SunRed, Shanghai, China; Catolog no. 106-201-12-8026). ELISA wells were read in a Biotek Instruments California, USA reader device. Glucose, TC, HDL-C, LDL-C, and TG tests were performed with a Beckman Coulter Brea, California, USA AU5800 chemistry autoanalyser following enzymatic methods. The insulin test was performed using a Beckman Coulter Brea, California USA DXI 800 immunoassay analyzer following the immunometric method. AMH tests were performed on a Siemens Healthineers Germany Advia Centaur XPT immunoassay analyzer following the immunometric method.

### 2.3. Statistical analysis

The required sample size was calculated using the G power program with an effect size of 0.56 [17], a power value of 80%, and an alpha-error value of 0.05. The number of subjects required for the study was 50 for each group. Descriptive statistical methods (mean, median, standard deviation, interquartile range) to evaluate data and the distribution of the variables were examined using the Shapiro-Wilk normality test. The Tukey multiple comparison test was used for subgroup comparisons. One-way ANOVA analysis of variance was used to compare the normally distributed variables between groups. Dunn’s multiple comparison test was used for subgroup comparisons. The Kruskal-Wallis test was used for the intergroup comparisons of the nonnormally distributed variables. The Pearson correlation test was used to determine the relationships between the variables. Results at the p < 0.05 value were accepted to be statistically significant. The Number Cruncher Statistical System 2007, Utah, USA (NCSS) package program was used for our evaluations.

## 3. Results

The mean age of the women in the obese PCOS group was 26.68 ± 4.55 years, 25.54 ± 4.74 years in the nonobese PCOS group, and 26.3 ± 4.4 years in the control group. The mean ages of all three groups were similar (p = 0.239). The BMI of the women in the obese PCOS group (36.05 ± 3.89) was statistically significantly higher than the BMI of the women in the nonobese PCOS group (26.58 ± 1.8) and the control group (26.92 ± 1.5) (p = 0.0001). A comparison of the demographic characteristics and biochemical parameters of the groups is given in Table 1. A statistically significant difference was found between serum sestrin 2 values of the women in the obese PCOS (6.42 ± 4.05 ng/mL), nonobese PCOS (8.19 ± 4.94 ng/mL) and control groups (15.18 ± 10.91 ng/mL) (p = 0.0001). The serum sestrin 2 values of the obese PCOS group were statistically significantly lower than the women in the nonobese PCOS and control groups (p = 0.001, p = 0.0001), while the sestrin 2 values of the nonobese PCOS group were statistically significantly lower than the women in the control group (p = 0.0001). A statistically significant difference was observed between the serum TRB3 values of the women in the obese PCOS (781.22 ± 476.75 pg/mL), non-obese PCOS (583.31 ± 362.46 pg/mL) and control groups (228.49 ± 170.76 pg/mL) (p = 0.0001). The serum TRB3 values of the control group were statistically significantly lower than those of the obese and nonobese PCOS groups (p = 0.0001), while the serum TRB3 values of the nonobese PCOS group were statistically significantly lower than those of the obese PCOS group (p = 0.0001) (Table 2). While the AMH values of the women in the control group were statistically significantly lower than all the women with PCOS (2.64 ± 1.18 ng/mL, p = 0.0001), there was no statistically significant difference between the AMH values of the women in the obese PCOS group (9.46 ± 3.21 ng/mL) and the AMH values of the women in the nonobese PCOS group (10.17 ± 4.32 ng/mL) (p = 0.395). A statistically significant positive correlation was observed between the sestrin 2 values and TRB3 values (r = 0.362, p = 0.0001), and between the TRB3 values and AMH values (r = 0.362, p = 0.0001). No statistically significant correlation was observed between the sestrin 2 values and AMH values (r = 0.05 p = 0.410).

A statistically negative significant correlation was observed between sestrin values and BMI (r = −0.272 p = 0.0001), insulin (r = −0.261 p = 0.0001) and HOMA (r = −0.250 p = 0.0001) values. No statistically significant correlation was observed between sestrin values and age, fasting plasma glucose, TG, TC, LDL-C and HDL-C values (p > 0.05). A statistically significant positive correlation was observed between serum TRB3 values and TG values (r = 0.248 p = 0.0001), and LDL-C values (r = 0.235 p = 0.0001). No statistically significant correlation was observed between TRB3 values and the other parameters (p > 0.05). A statistically positive significant correlation was observed between AMH values and BMI (r = 0.233 p = 0.0001), insulin (r = 0.253 p = 0.0001), HOMA (r = 0.266 p = 0.0001), TG (r = 0.248 p = 0.0001) and LDL-C (r = 0.332, p = 0.0001) values. No statistically significant correlation was observed between AMH values and age, fasting plasma glucose, TC, and HDL-C values (p > 0.05) (Table 3).

## 4. Discussion

In this study, serum sestrin 2 levels were statistically significantly lower and serum TRB3 levels were statistically significantly higher in the obese PCOS group compared to the control and nonobese PCOS groups. While there are many studies in the literature examining the relationship between sestrin 2 and TRB3 with various diseases [23**–**26], no study has been found examining its relationship with obesity in women with PCOS. Type 2 diabetes, cardiovascular disease, dyslipidemia, fatty liver and obstructive sleep apnea are seen 2–3 times more frequently in women with PCOS with high insulin and BMI values compared to normal women [27]. Moreover, disruption of the balance between free oxygen radicals and antioxidants in PCOS causes exposure to oxidative stress.

The expression of sestrin 2 increases under conditions of stress with sestrin 2 protecting cells against oxidative stress [28]. In a study in mouse models, it was reported that sestrin 2 may be a therapeutic target due to its protective effect on hepatocytes [29]. Studies have shown that obesity is related to decreased antioxidant capacity and increased levels of reactive oxygen species [23,30]. Moreover, decreases in endogen sestrin 2 levels may be related to a number of metabolic disturbances, such as insulin resistance and fat accumulation [31]. It has been shown that the risk of coronary artery disease and atherosclerosis increases if sestrin 2 levels are not sufficiently elevated [32,33]. Xu et al. showed that in women with PCOS, when the level of sestrin 1 decreases, autophagy decreases and free oxygen radicals increase [34]. Sestrin 2 inhibits mammalian target of rapamycin (MTORC) and activates the adenosine monophosphate-activated protein kinase (AMPK), the main regulator of energy balance; thus, it has a crucial role in protecting against obesity [35,36]. Disruption of MTORC1 signaling may result in abnormal folliculogenesis [37]. We believe that the negative effects of PCOS on metabolic parameters might decrease sestrin 2 levels. Thus, inactivated AMPK may cause obesity. Moreover, the higher levels of metabolic markers, which were present in the obese PCOS group, such as fasting plasma glucose, insulin, HOMA-IR and TG, confirm this relationship in our study. However, inconsistencies are found in the studies. In a recent study on women with PCOS, plasma sestrin 2 levels were shown to be significantly lower than in healthy controls [38], and higher in another study [16]. In this study, nonobese and obese women with PCOS were separated and compared in terms of sestrin 2 levels. It was concluded that high levels of sestrin 2 in nonobese PCOS patients may be related to the high relative antioxidant capacity of this patient group. The group with low sestrin 2 levels, that is, the obese PCOS group, may be inclined to be overweight because they cannot show this capacity sufficiently. As a matter of fact, studies that found an increase in serum sestrin 2 in individuals with metabolic syndrome, as in this study, explained this paradoxical increase as a compensatory mechanism to overcome metabolic stress [39**–**41]. Our results also support these findings, as we observed that sestrin 2 levels were lower and metabolic parameters were impaired in the overweight PCOS group.

In this study, TRB3 levels were found to be higher in both the nonobese group and obese PCOS group compared to the control group. Nourbakhsh et al. also showed that obese children had significantly lower sestrin 2 concentrations and higher TRB3 than normal-weight children [17]. These findings for TRB3 and sestrin 2 are consistent with reports previously mentioned in the literature. TRB3 plays an important role in glucose and lipid metabolism. Moreover, it becomes active in adipocytes, which are differentiated when metabolic syndrome and obesity occur. It is shown that the overexpression of TRB3 increases glucose exit from the liver in mouse models and brings those mice into a diabetic state [17]. Another finding confirming this idea is the positive correlation between BMI and TG levels in the nonobese PCOS group. In addition, according to our findings, high AMH levels in all groups with PCOS have been described in the literature as extremely strong. It is described in the literature that high levels of AMH occur in PCOS patients who have insulin resistance [42]. Statistically very strong elevations of TRB3 and AMH in all PCOS groups may indicate a relationship between insulin resistance, impaired metabolic parameters, obesity and PCOS. In this study, it was demonstrated that impaired regulation of TRB3 in PCOS patients may contribute to obesity and associated metabolic disorders. On the other hand, low levels of sestrin 2 in the obese PCOS group may be considered a risk factor for obesity and metabolic disorders.

There are a number of limitations in this study such that the current research was limited by the small sample size. Therefore, we could not form a separate overweight group (BMI = 25–30 kg/m^2^). Further limitations include ignoring smoking status, not examining the oxidative stress parameters and the visceral adiposity index.

In conclusion, according to the findings in this study, low sestrin 2 and high TRB3 levels may be related to impaired metabolic status in the obese PCOS group. Metabolic stress may cause a decrease in sestrin 2 levels in the obese PCOS group. The protective role of sestrin 2 can be explained by the negative correlation of sestrin 2 with metabolic markers in the nonobese PCOS group. We surmise that the high levels of TRB3 in both PCOS groups may be related to metabolic homeostasis disorders caused by PCOS and obesity. When the effects of TRB3 on glucose metabolism are considered to be related to obesity, we believe that TRB3 may be a parameter predicting the occurrence of obesity, which is more common in PCOS patients. As a result, sestrin 2 and TRB3 may be suggested as biomarkers that can predict the development of obesity in patients with PCOS. However, it is unclear whether obesity develops due to insufficient antioxidant response in PCOS or whether obesity inhibits the antioxidant response. Treatments that activate the sestrin 2 level and target to inhibit the TRB3 level may shed light on the development of new treatment modalities in PCOS patients. However, further research is needed to determine the precise role of these proteins in the pathophysiology of PCOS.

## Figures and Tables

**Table 1:** Biochemical characteristics of the groups.

	Obese PCOS group (n = 90) Mean ± SD	Non-obese PCOS group (n = 90) Mean ± SD	Control group (n = 90) Mean ± SD	p value
**Age** (years)	26.68 ± 4.55	25.54 ± 4.74	26.3 ± 4.4	0.239
**BMI** (kg/m^2^)	36.05 ± 3.89	26.58 ± 1.8	26.92 ± 1.5	**0.0001**
**Fasting plasma glucose** (mg/dL)	95.98 ± 6.11	87.54 ± 6.6	88.52 ± 7.17	**0.0001**
**Insulin** (μIU/mL)	11.74 ± 2.81	6.91 ± 1.87	6.44 ± 1.38	**0.0001**
**HOMA-IR**	2.71 ± 0.69	1.43 ± 0.44	1.3 ± 0.33	**0.0001**
**TG** (mg/dL)	107.11 ± 24.9	95.59 ± 18.93	73.06 ± 18.06	**0.0001**
**TC** (mg/dL)	164.56 ± 25.72	166.59 ± 28.85	156.57 ± 19.91	**0.019**
**LDL-C** (mg/dL)	76.44 ± 10.96	88.88 ± 16.56	69.2 ± 11.47	**0.0001**
**HDL-C** (mg/dL)	51.05 ± 8,51	49.71 ± 8.06	51.12 ± 7.81	0.421
**AMH** (ng/mL)	9.46 ± 3.21	10.17 ± 4.32	2.64 ± 1.18	**0.0001**

SD: Standard deviation, p < 0.05,

PCOS: Polycystic ovary syndrome, BMI: Body Mass Index, HOMA-IR: Homeostatic model assessment of IR, TG: Triglycerides, TC: Total cholesterol, LDL-C: Low density lipoprotein cholesterol, HDL-C: High density lipoprotein-cholesterol, AMH: Anti-Mullerian hormone.

**Table 2:** Comparison of serum sestrin 2 and tribbles homolog 3 levels of the groups.

	Obese PCOS group (n = 90)	Non-obese PCOS group (n = 90)	Control group (n = 90)	p value

**Sestrin 2** (ng/mL) Mean ± SD	6.42 ± 4.05	8.19 ± 4.94	15.18 ± 10.91	**0.0001**
Median (IQR)	5.29 (4.25–7.55)	7.22 (5.09–9.78)	9.18 (7.06–1.29)	

**TRB3** (pg/mL) Mean ± SD	781.22 ± 476.75	583.31 ± 362.46	228.49 ± 170.76	**0.0001**
Median (IQR)	608.67 (497.5–900.65)	489.26 (363.63–636.25)	165 (93.5–342)	

IQR: Interquartile range, p < 0.05

PCOS: Polycystic ovary syndrome, TRB3: Tribbles homolog 3

**Table 3:** Relationships between sestrin 2, TRB3 and AMH values with biochemical parameters.

		Sestrin 2	TRB3	AMH
**Age**	r	−0.169	−0.021	0.034
	p	0.005	0.732	0.583
**BMI**	r	**−0.272**	0.066	**0.233**
	p	**0.0001**	0.279	**0.0001**
**Fasting plasma glucose**	r	−0.06	0.096	0.091
	p	0.327	0.116	0.136
**Insulin**	r	**−0.261**	0.130	**0.253**
	p	**0.0001**	0.034	**0.0001**
**HOMA-IR**	r	**−0.250**	0.143	**0.266**
	p	**0.0001**	0.019	**0.0001**
**TG**	r	0.022	**0.248**	**0.248**
	p	0.719	**0.0001**	**0.0001**
**TC**	r	0.101	0.062	0.083
	p	0.098	0.315	0.172
**LDL-C**	r	0.181	**0.235**	**0.332**
	p	0.003	**0.0001**	**0.0001**
**HDL-C**	r	0.026	−0.085	−0.036
	p	0.673	0.164	0.554

r: Correlation coefficient, p < 0.05

BMI: Body Mass Index, HOMA-IR: Homeostatic model assessment of IR, TG: Triglycerides, TC: Total cholesterol, LDL-C: Low-density lipoprotein cholesterol, HDL-C: High-density lipoprotein-cholesterol, AMH: Anti-Mullerian hormone.
